# Effect of intravenous immunoglobulin therapy on the prognosis of patients with severe fever with thrombocytopenia syndrome and neurological complications

**DOI:** 10.3389/fimmu.2023.1118039

**Published:** 2023-03-22

**Authors:** Yun Liu, Hanwen Tong, Fei He, Yu Zhai, Chao Wu, Jun Wang, Chenxiao Jiang

**Affiliations:** ^1^ Department of Emergency Medicine, Nanjing Drum Tower Hospital, The Affiliated Hospital of Nanjing University Medical School, Nanjing, China; ^2^ Department of Infectious Disease, Nanjing Drum Tower Hospital, The Affiliated Hospital of Nanjing University Medical School, Nanjing, China; ^3^ Department of Pharmacy, Nanjing Drum Tower Hospital, The Affiliated Hospital of Nanjing University Medical School, Nanjing, China

**Keywords:** intravenous immunoglobulin, mortality, severe fever with thrombocytopenia syndrome, neurological complications, dosage, duration

## Abstract

**Background:**

Intravenous immunoglobulin (IVIG) has been reported to exert a beneficial effect on severe fever with thrombocytopenia syndrome (SFTS) patients with neurological complications. However, in clinical practice, the standard regime is unclear and there is a lack of evidence from large-scale studies.

**Methods:**

A single-center retrospective study was conducted to determine the influence of IVIG dosage and duration on SFTS patients with neurological complications. The primary outcome was 28-day mortality, and laboratory parameters before and after IVIG treatment were measured. Survival curves were generated using the Kaplan–Meier method and analyzed with the log-rank test according to the median IVIG dosage and IVIG duration. Besides, multivariate Cox regression analysis was performed to examine the association between the independent factors and 28-day mortality in SFTS patients.

**Results:**

Overall, 36 patients (58.06%) survived, while 26 (41.9%) patients died. The median age of the included patients was 70 (55–75) years, and 46.8% (29/62) were male. A significantly higher clinical presentation of dizziness and headache was observed in the survival group. The IVIG duration in the survival group was longer than in the death group (*P <*0.05). Additionally, the IVIG dosage was higher in the survival group than in the death group, but there was not a statistically significant difference between the two groups (*P* = 0.066). The mediating effect of IVIG duration was verified through the relationship between IVIG dosage and prognosis using the Sobel test. Univariate analysis revealed that IVIG dosage (HR: 0.98; 95% CI: 0.97–1.00; *P* = 0.007) and IVIG duration (HR: 0.54; 95% CI: 0.41–0.72; *P <*0.001) were significantly associated with risk of death. The multivariate analysis generated an adjusted HR value of 0.98 (95% CI: 0.96–1.00; *P* = 0.012) for IVIG dosage and 0.26 (95% CI: 0.09–0.78; *P* = 0.016) for dizziness and headache.

**Conclusion:**

Prolonged high-dose IVIG is beneficial to the 28-day prognosis in SFTS patients with neurological complications.

## Introduction

Severe fever with thrombocytopenia syndrome (SFTS) is a potentially fatal tick-borne infectious disease caused by the SFTS virus (SFTSV). It has a mortality rate ranging from 5% to over 40% and an average mortality rate of 12.2% in Asia (1, 2). To date, SFTS treatment mainly relies on supportive care and the most widely used therapy is ribavirin. However, its efficacy still requires further verification (3–5). In clinical practice, the occurrence of nervous system symptoms is a predictor of poor prognosis (6, 7). SFTS-related neurological complications specifically refer to the following symptoms (1): involuntary muscle tremors of the tongue, jaw, or extremities (2); cognitive deficits (3); consciousness-related problems, including drowsiness, lethargy, coma, and delirium (4); convulsions or tics (8). Moreover, we believe drugs that can resolve nervous system symptoms may be beneficial for such patients. Several case reports have demonstrated the efficacy of intravenous immunoglobulin (IVIG) and corticosteroids in treating SFTS with neurological complications (9, 10). However, since steroid administration may be hazardous to SFTS patients with fungal infections (11, 12), IVIG appears to be a more safe and effective option for SFTS treatment. Various studies have revealed that IVIG treatment is effective in curing viral encephalitis (13), suppressing cytokine storms (14), and treating several autoimmune or inflammatory neurological diseases such as Guillain–Barre syndrome (GBS), chronic inflammatory demyelinating polyneuropathy (CIDP), multifocal motor neuropathy (MMN), and myasthenia gravis (MG) (15). Thus, from a mechanical perspective, IVIG may be useful for treating SFTS-related neurological symptoms and we recommend that clinicians initiate IVIG treatment through a standard treatment protocol, especially for patients with neurological symptoms. However, due to its high cost, it is important to optimize the clinical value of IVIG. For most patients, a regimen of 400 mg/kg with a duration of 3–5 days is recommended but the dosage and duration vary in practice (16). In this paper, we discuss the effect of IVIG dosage and duration on the prognosis of SFTS patients with neurological symptoms. Ultimately, we determined that an IVIG dosage of more than or equal to 80 g through a prolonged treatment duration of five or more days serves as a good prognosis predictor in SFTS with neurological symptoms. Thus, this provides a basic guideline for IVIG treatment in clinical practice.

## Materials and methods

### Study population

In this retrospective study, we reviewed the files of 223 SFTS patients who were admitted to Nanjing Drum Tower Hospital between 2014 and 2022. Our study was approved by the Ethics Committee of the hospital. Besides, the committee waived the requirement for written informed consent considering the retrospective nature of the study. The inclusion criteria were patients diagnosed with SFTS according to the following two conditions (1): Acute fever over 38°C with thrombocytopenia (platelet (PLT) count <100 × 10^9^/L) (2); Laboratory-confirmed SFTSV infection using a certified real-time polymerase chain reaction (RT-PCR) kit (17). The exclusion criteria were (1): Patients who had not received IVIG therapy since STFS occurred (2); Patients who presented no neurological symptoms throughout the course of STFS.

After admission, all patients received ribavirin (0.5 g q12 h) until their temperature improved, they were discharged automatically, or they died. Symptomatic and supportive therapies, including antibacterial therapy, antifungal therapy, corticosteroid therapy, recombinant human granulocyte colony-stimulating factor (rhG-CSF) therapy, plasma exchange, blood transfusion, oxygen inhalation, and respiratory support (non-invasive or invasive ventilation), were administered according to the symptoms of the patients and the judgment of the physicians.

### Clinical data collection

Clinical data were collected from the electronic medical record system in Nanjing Drum Tower Hospital, including demographic information, chronic comorbidities, clinical manifestations, and IVIG usage, which was expressed as IVIG dosage and duration. Specifically, IVIG dosage corresponds to the cumulative IVIG dosage, while IVIG duration is defined as the number of days of IVIG treatment. Laboratory parameters were obtained before and after IVIG treatment (within two days of concluding IVIG treatment). Follow-up was defined as the 28th day from the start of IVIG treatment, while the primary outcome was 28-day mortality.

### Statistical analysis

Demographic and clinical data were analyzed using STATA v.17.0 (Stata Corp LLC, USA). Normally distributed continuous variables were expressed as mean ± standard deviation or median and interquartile range (IQR). Comparisons of continuous variables were conducted using the t-test or Mann–Whitney U test. Meanwhile, comparisons of categorical variables expressed as frequency and percentage (%) were performed using the chi-square test or Fisher exact test. Besides, comparisons of laboratory parameters before and after IVIG treatment were carried out with the paired sample t-test or Wilcoxon signed-rank test. Survival curves were generated with the Kaplan–Meier method and analyzed using the log-rank test according to the median IVIG dosage or IVIG duration. Univariate and multivariate Cox regression analyses examined the relationship between independent factors and 28-day mortality in SFTS patients. A Sobel test was performed to evaluate the mediating role of IVIG duration in the relationship between IVIG dosage and prognosis. The results of the analyses were reported as hazard ratios (HRs) with 95% confidence intervals (CIs). Also, a value of *P <*0.05 was considered statistically significant.

## Results

**Figure 1:**
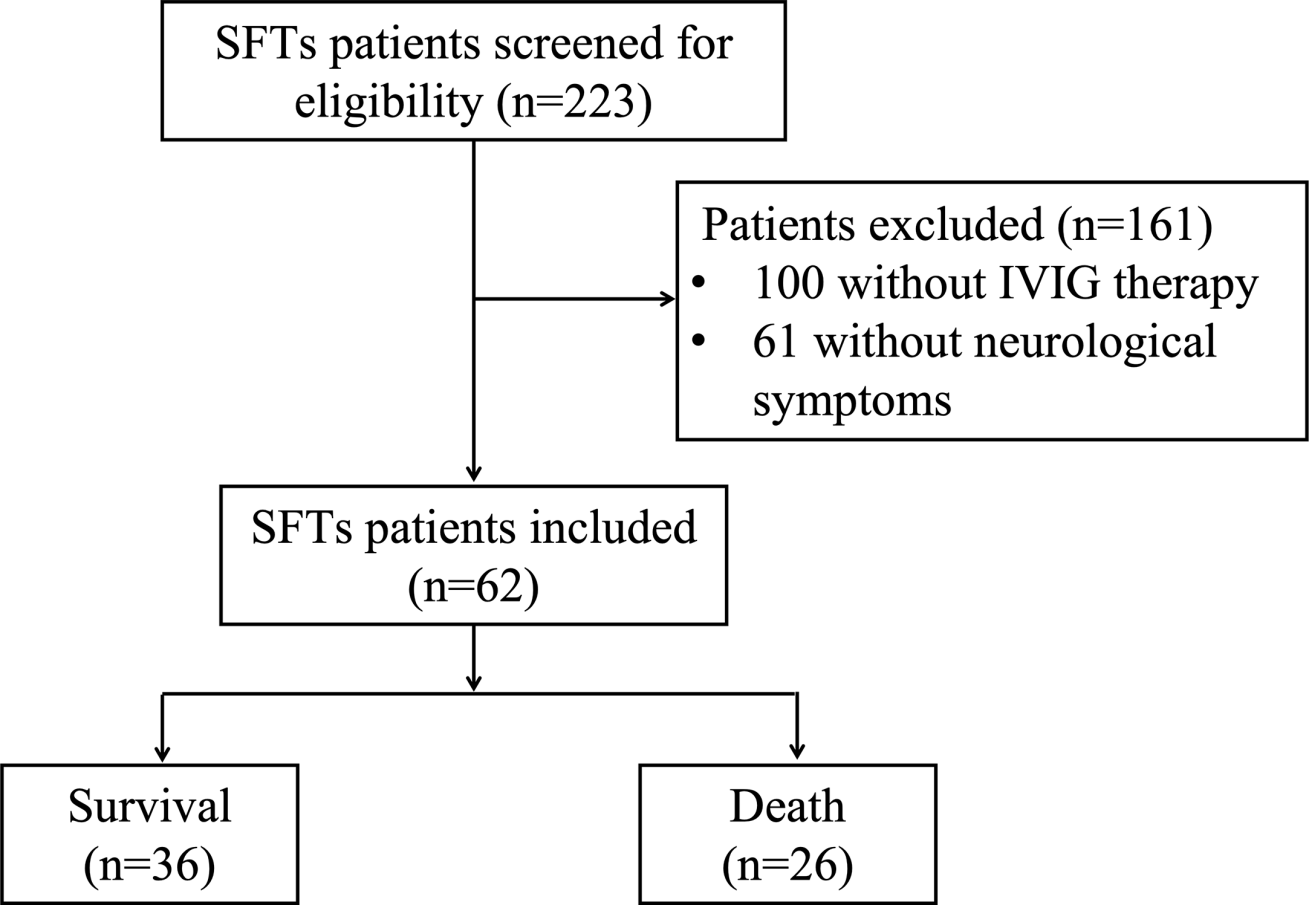
Research flowchart. SFTS nucleic acid tests were performed in the general or emergency clinic. The dosage and treatment duration and neurological symptoms of the patients were recorded. During the first and second weeks of hospitalization, routine blood, biochemical, urine, coagulation, and inflammatory indexes were checked daily. During the third and fourth weeks of hospitalization, laboratory tests were performed according to the condition of the patients.

In this study, a total of 223 SFTS patients were screened for eligibility. Of these patients, 161 were excluded, while 62 patients with neurological symptoms who also received IVIG treatment were included (**Figure 1**). Of the 62 patients in our study with neurological disorders, four (6.5%) had limb tremors, 13 (21.0%) experienced lethargy, five (8.1%) were unresponsive, 11 (17.7%) felt agitation, 24 (38.7%) suffered from confusion, eight (12.9%) felt drowsiness, six (9.7%) had delirium, eight (12.9%) were comatose, and two (3.2%) suffered from convulsions. Six patients underwent cerebrospinal fluid (CSF) tests, which revealed increased levels of protein and glucose. Additionally, an electroencephalogram was administered to eight patients, seven of whom presented moderate abnormalities.

**Figure 2:**
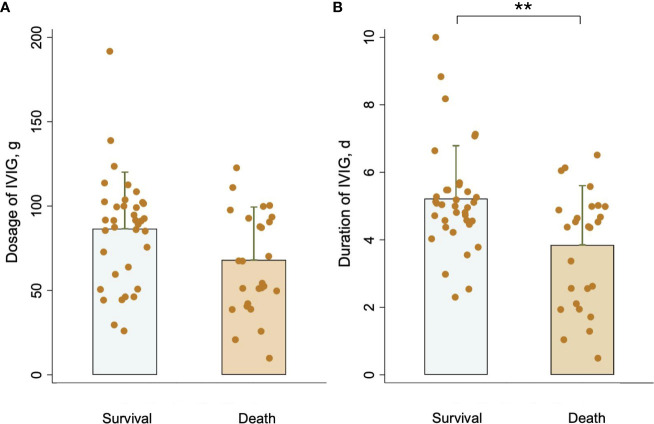
Comparison of **(A)** IVIG dosage and **(B)** IVIG duration between the survival group and the death group. **P <0.01.

**Table 1** presents baseline clinical characteristics and laboratory findings for patients in the survival group and the death group. A substantially higher clinical presentation of dizziness and headache was observed in the survival group. Besides, the death group exhibited considerably higher baseline laboratory data values for absolute lymphocyte count (ALC), activated partial thromboplastin time (APTT), and D-dimer, which were consistent with other reports. Moreover, IVIG duration in the survival group was longer than in the death group (mean: 5 ± 2 vs 4 ± 2, *P <*0.05). Additionally, IVIG dosage was higher in the survival group than in the death group, but there was no significant difference between the two groups [median: 95.0 (57.5–100.0) vs 60.0 (40.0–100.0), *P* = 0.066]. **Figure 2** compares the IVIG dosage and duration between the survival and death groups. According to **Figure 2A**, the median IVIG dosage in the survival group was higher than in the death group, but with no statistical difference. Moreover, **Figure 2B** indicates that the median IVIG duration in the survival group was significantly longer than that in the death group.

**Table 1:** Baseline clinical characteristics and laboratory parameters of patients in the survival and death groups.

Variable	Survival (n = 36)	Death (n = 26)	P-value
Demographics			
Age, years	67 (54–73)	71 (58–76)	0.199
Male, n (%)	18 (50.0%)	11 (42.3%)	0.549
Chronic comorbidities, n (%)			
Hypertension	11 (30.6%)	10 (38.5%)	0.516
Diabetes mellitus	3 (8.3%)	2 (7.7%)	1.000
Malignancy	1 (2.8%)	0 (0%)	1.000
CAD	1 (2.8%)	0 (0%)	1.000
COPD	0 (0%)	1 (3.9%)	0.419
Clinical manifestations, n (%)			
Nausea	12 (33.3%)	7 (26.9%)	0.589
Vomiting	10 (27.8%)	9 (34.6%)	0.564
Celialgia	4 (11.1%)	2 (7.7%)	0.653
Diarrhea	17 (47.2%)	10 (38.5%)	0.492
Unintelligible speech	1 (2.8%)	3(11.5%)	0.300
Dizziness and headache	18 (50.0%)	6 (23.1%)	0.032
Cough	9 (25.0%)	4 (15.4%)	0.359
Sputum	7 (19.4%)	3 (11.5%)	0.627
Chest tightness	3 (8.3%)	3 (11.5%)	0.689
Rash	6 (16.7%)	1 (3.8%)	0.222
Lymphadenopathy	13 (36.1%)	7 (26.9%)	0.445
Bleeding spots on the skin	7 (19.4%)	9 (34.6%)	0.178
Laboratory parameters			
WBC count, median (IQR), ×10^9^/L	2.5 (1.8–4.6)	2.7 (1.7–3.7)	0.898
ANC count, median (IQR), ×10^9^/L	1.7 (1.0–2.9)	2.1 (1.2–2.8)	0.668
ALC count, median (IQR), ×109/L	0.7 (0.4–0.9)	0.5 (0.3–0.6)	0.042
NLR, median (IQR)	2.2 (1.5–6.1)	3.3 (1.9–9.1)	0.136
RDW, median (IQR), %	13.2 (12.8–13.5)	13.6 (12.8–14.3)	0.071
PLT count, median (IQR), ×10^9^/L	44.5 (33.8–61.3)	39.5 (29.0–58.5)	0.480
PLR, median (IQR)	65.5 (39.3–146.0)	89.6 (67.5–145.0)	0.248
PT, median (IQR), s	12.0 (11.2–12.6)	12.4 (11.7–13.0)	0.123
APTT, median (IQR), s	41.4 (33.6–46.3)	48.5 (37.6–61.1)	0.009
TT, median (IQR), s	22.8 (21.1–27.7)	26.9 (21.8–57.9)	0.057
D-dimer, median (IQR), mg/L	3.8 (2.3–9.2)	9.9 (3.4–22.6)	0.018
ALT, median (IQR), U/L	69.6 (54.3–97.2)	75.3 (50.5–90.5)	0.881
AST, median (IQR), U/L	172.5 (93.4–312.9)	222.0 (114.1–321.8)	0.304
ALB, median (IQR), g/L	32.0 (28.7–35.7)	31.7 (29.1–34.1)	0.775
TBIL, median (IQR), µmol/L	11.0 (8.6–13.9)	7.7 (5.8–11.5)	0.066
SCr, median (IQR), µmol/L	72.5 (48.8–89.6)	82 (67.5–123.6)	0.090
BUN, median (IQR), mmol/L	5.2 (3.5–7.1)	5.9 (4.8–10.6)	0.061
UA, median (IQR), µmol/L	306 (236–347)	342 (231–463)	0.471
IVIG usage			
IVIG dosage, g	95.0 (57.5–100.0)	60.0 (40.0–100.0)	0.066
IVIG duration, d	5 ± 2	4 ± 2	0.003
Additional information			
Time from onset to arriving in hospital, d	7 ± 3	7 ± 2	0.317
Time from onset to IVIG treatment, d	9 ± 3	9 ± 3	0.677

CAD, coronary artery disease; COPD, chronic obstructive pulmonary disease; WBC, white blood cell; ANC, absolute neutrophil count; ALC, absolute lymphocyte count; NLR, neutrophil-to-lymphocyte ratio; RDW, red cell volume distribution width; PLT, platelet; PLR, platelet-to-lymphocyte ratio; PT, prothrombin time; APTT, activated partial thromboplastin time; TT, thrombin time; ALT, alanine aminotransferase; AST, aspartate aminotransferase; ALB, serum albumin; TBIL, total bilirubin; SCr, serum creatinine; BUN, blood urea nitrogen; UA, uric acid; IVIG, intravenous immunoglobulin; IQR, interquartile range.

Additionally, **Table 1** lists therapies other than IVIG in the survival and death groups. Patients in the death group received higher rates of corticosteroid therapy, blood transfusions, oxygen therapy, and respiratory support (*P <*0.05).

**Figure 3:**
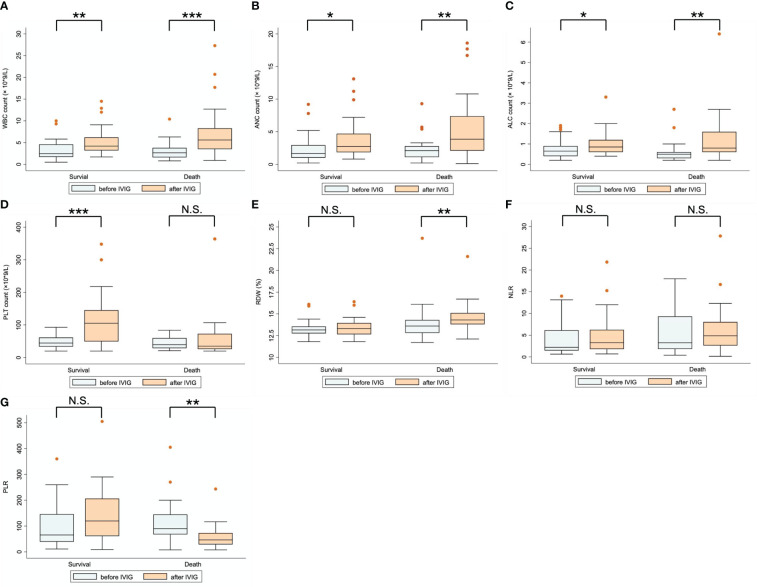
Comparison of laboratory parameters before and after IVIG treatment for patients in the survival and death groups. **(A)** WBC count; **(B)** ANC count; **(C)** ALC count; **(D)** PLT count; **(E)** RDW; **(F)** NLR; **(G)** PLR. *P < 0.05; **P < 0.01; ***P < 0.001; N.S., represents no significant difference.

**Table 2** compares the laboratory parameters of SFTS patients before and after IVIG treatment. White blood cell count (WBC), absolute neutrophil count (ANC), absolute lymphocyte count (ALC), red cell volume distribution width (RDW), and PLT count were substantially higher after IVIG treatment than before IVIG treatment. In contrast, the neutrophil-to-lymphocyte ratio (NLR) and the platelet-to-lymphocyte ratio (PLR) exhibited no differences. **Figure 3** illustrates the changes in laboratory parameters before and after IVIG administration in the survival group and the death group. WBC, ANC, and ALC were much higher in the death group than in the survival group after IVIG treatment (**Figures 3A–C**). In the survival group, the PLT count was significantly higher after IVIG treatment (*P <*0.001), while in the death group, it showed no significant difference before and after IVIG treatment (**Figure 3D**). Besides, in the death group, RDW was slightly higher after IVIG treatment *(P <*0.01). In contrast, no significant difference in RDW was observed in the survival group before and after IVIG treatment (**Figure 3E**). Furthermore, NLR was not significantly different before or after IVIG treatment in either the survival or the death group (**Figure 3F**). Nevertheless, PLR decreased significantly after IVIG treatment in the death group (*P <*0.01), but it did not differ appreciably between pre-IVIG and post-IVIG treatment in the survival group (**Figure 3G**).

**Table 2:** Comparison of laboratory parameters in SFTS patients before and after IVIG treatment.

Variable	Before IVIG	After IVIG	P-value
WBC count, median (IQR), ×10^9^/L	2.6 (1.6–4.3)	4.9 (3.4–7.4)	<0.001
ANC count, median (IQR), ×10^9^/L	1.8 (1.1–2.9)	3.2 (2.0–6.1)	<0.001
ALC count, median (IQR), ×10^9^/L	0.5 (0.4–0.7)	0.8 (0.6–1.2)	<0.001
NLR, median (IQR)	2.6 (1.6–6.5)	3.8 (2.0–6.8)	0.318
RDW, median (IQR), ×10^9^/L	13.2 (12.8–14.0)	13.8 (12.9–14.4)	<0.001
PLT count, median (IQR), ×10^9^/L	43.0 (32.2–60.0)	72.0 (31.8–122.8)	<0.001
PLR, median (IQR)	81.3 (41.7–145.0)	70.7 (38.8–163.3)	0.940

WBC, white blood cell; ANC, absolute neutrophil count; ALC, absolute lymphocyte count; NLR, neutrophil-to-lymphocyte ratio; RDW, red cell volume distribution width; PLT, platelet; PLR, platelet-to-lymphocyte ratio; IVIG, intravenous immunoglobulin; IQR, interquartile range.

The association between IVIG dosage and duration with 28-day mortality in SFTS patients was further investigated using univariate and multivariate Cox regression analysis, as **Table 3** shows. The univariate analysis revealed that IVIG dosage (HR: 0.98; 95% CI: 0.97–1.00; *P* = 0.007), duration of IVIG treatment (HR: 0.54; 95% CI: 0.41–0.72; *P <*0.001), APTT (HR: 1.02; 95% CI: 1.00–1.04; *P* = 0.014), TT (HR: 1.02; 95% CI: 1.01–1.03; *P* = 0.002), D-dimer (HR: 1.04; 95% CI: 1.01–1.07; *P* = 0.012), blood transfusion (HR: 11.31; 95% CI: 1.53–83.62; *P* = 0.017), oxygen inhalation (HR: 3.77; 95% CI: 1.13–12.58; *P* = 0.031), and respiratory support (HR: 2.85; 95% CI: 1.27–6.44; *P* = 0.011) were all significantly associated with risk of death. 100% of the indirect effect (mediation effect) of IVIG duration on SFTS patient prognosis was observed (Sobel *P* = 0.028; **Figure 1**; **Table 2**). Thus, IVIG duration was excluded from the multivariate Cox analysis. In the multivariate analysis, the adjusted HR was 0.98 (95% CI: 0.96–1.00; *P* = 0.012) for IVIG dosage and 0.26 (95% CI: 0.09–0.78; *P* = 0.016) for dizziness and headache.

**Table 3:** Independent variables associated with 28-day mortality in SFTS patients according to univariate and multivariate Cox regression analyses.

Independent Variable	Univariate analysis		Multivariate analysis	
	HR (95% CI)	P-Value	HR (95% CI)	P-Value
IVIG dosage	0.98 (0.97–1.00)	0.007	0.98 (0.96–1.00)	0.012
IVIG duration	0.54 (0.41–0.72)	<0.001		
NLR	1.07 (0.99–1.16)	0.111		
RDW	1.14 (0.96–1.35)	0.136		
PT	1.44 (0.97–2.13)	0.069	1.27 (0.72–2.24)	0.415
APTT	1.02 (1.00–1.04)	0.014	0.99 (0.96–1.03)	0.591
TT	1.02 (1.01–1.03)	0.002	1.01 (0.99–1.02)	0.315
D-dimer	1.04 (1.01–1.07)	0.012	1.00 (0.96–1.04)	0.972
Dizziness and headache	0.42 (0.17–1.05)	0.063	0.26 (0.09–0.78)	0.016
Corticosteroid therapy	1.91 (0.85–4.29)	0.118		
RhG-CSF therapy	2.15 (0.74–6.24)	0.160		
Plasma exchange	2.38 (0.81–6.94)	0.115		
Blood transfusion	11.31 (1.53–83.62)	0.017	7.16 (0.90–57.35)	0.064
Oxygen inhalation	3.77 (1.13–12.58)	0.031	2.27 (0.54–9.58)	0.264
Respiratory support	2.85 (1.27–6.44)	0.011	2.26 (0.80–6.44)	0.126

IVIG dosage represents the cumulative dosage of IVIG; IVIG duration is defined as the number of days of IVIG treatment; IVIG, intravenous immunoglobulin; NLR, neutrophil-to-lymphocyte ratio; RDW, red cell volume distribution width; PT, prothrombin time; APTT, activated partial thromboplastin time; TT, thrombin time; RhG-CSF, recombinant human granulocyte colony-stimulating factor; HR, hazard ratio; CI, confidence interval.

**Figure 4:**
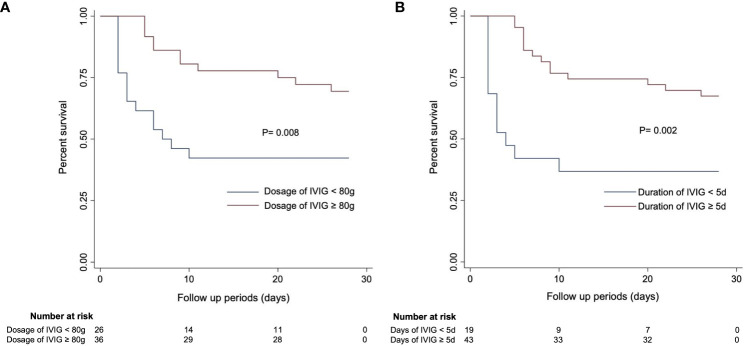
Kaplan–Meier curves estimating 28-day mortality in SFTS patients based on **(A)** IVIG dosage and **(B)** IVIG duration.

Additionally, the Kaplan-Meier (K-M) curves (**Figure 4**) revealed that there were differences in survival outcomes according to IVIG dosage and duration. Patients with an IVIG dosage of more than or equal to 80 g (**Figure 4A**) and an IVIG duration of 5 days or more (**Figure 4B**) had higher survival rates.

## Discussion

Although the pathogenesis of SFTS has not been conclusively established, it is widely believed that the immune system plays a vital role in the infection process. Decreased levels of CD3+ and CD4+ T cells and increased natural killer T (NKT) cell activity were observed in the acute phase of the SFTS process (18). Increased NKT cell activity is closely related to the release of multiple cytokines such as interferon-γ, interleukin-10, and granulocyte colony-stimulating factor. Additionally, reduced CD4+ T-cell levels are associated with lower immunoglobulin levels, which may lead to immunosuppression. Besides, several studies have revealed that cytokine storms are a major contributor to the pathogenesis of SFTS (19). Meanwhile, the pathogenesis of SFTS-associated nervous system symptoms is believed to be related to direct virus invasion, cytokine storms, and immune mediation (20–22). Also, the appearance of neurological manifestations may indicate that the cytokine storm has reached a peak. In our study, the patients generally developed neurological complications during the multiple organ dysfunction syndrome (MODS) period, which refers to the period 7–13 days from onset. Moreover, IVIG was administrated on around the ninth day. Previous reports revealed that approximately 19.1% of patients infected with SFTSV developed encephalopathy and the fatality rate reached 44.7%, compared with 9.4% in non-encephalopathy patients (6). In our study, the fatality rate was 41.9% in SFTS patients with neurological complications. Therefore, drugs that alleviate viral infections and cytokine storms may be effective in treating certain patients, while immunomodulatory therapy may be considered an alternative therapy for SFTS treatment.

IVIG is a blood product that is extracted from the mixed plasma of healthy people. It is rich in bacterial antibodies and viral IgG and has been used for over 30 years. However, the mechanisms by which IVIG works are still not fully understood. Several components of IVIG are efficacious, including the F(ab) 2 variable region, which may inhibit proliferation by inducing apoptosis and inhibiting the cell cycle. IVIG also contains natural IgG antibodies with pathogenic and immunoregulatory molecules that reduce cytokine levels (23). Another vital component is the range of Fc receptors (FcR) in the Fc region that enhance the catabolism of endogenous IgG and reduce autoantibody levels in some models (24). IVIG has been proven to inhibit macrophage and cytokine storms in cases of Crimean–Congo hemorrhagic fever (14), which is similar to SFTS in terms of viral characteristics, disease manifestations, and pathophysiology (1). Clinically, IVIG is recommended as first-line therapy in the treatment of acute and chronic neuropathy (15). Meanwhile, several studies have verified the effectiveness of IVIG treatment in illnesses involving the central nervous system and encephalitis (13, 25). Thus, IVIG is believed to be effective in SFTS treatment, especially in patients with neurological complications. Besides, a full recovery from abnormal neurological manifestations was observed in several SFTS patients after IVIG treatment (9). Concerning laboratory parameters, previous studies demonstrated that decreased levels of PLT, ALC, and MON% (monocyte percentage) were strongly associated with an increased risk of death in SFTS patients. However, WBC was not associated with SFTS outcomes, although leukopenia was a typical feature of SFTS (26, 27). NLR (neutrophil-to-lymphocyte ratio) and PLR (platelet-to-lymphocyte ratio) are regarded as prognosis indicators in SFTS and an increased NLR is connected to a greater risk of death in SFTS patients (28, 29). In our study, significantly higher levels of WBC, ANC, ALC, and RDW were observed in the death group after IVIG treatment. Additionally, PLT levels were substantially higher in the survival group after IVIG treatment, while PLR was much lower in the death group after IVIG. This was mainly the result of the change in PLT count, which suggests that dynamically monitoring PLT during IVIG treatment may predict the prognosis of SFTS patients with neurological symptoms.

Studies have revealed that IVIG administration is relatively safe with few side effects. Complications of IVIG therapy are mild and transitory and include fever, fatigue, chills, malaise, headache, nausea, diarrhea, dyspnea, back pain, and tachycardia. These conditions only occur during the early stages of IVIG administration and are resolved within a few days. However, due to its high cost, some patients refuse IVIG treatment. Thus, it is crucial to optimize its use, especially for SFTS patients, who generally come from less affluent rural areas. Since research has proved that IVIG is mechanically and clinically effective, proper protocols should be devised to guarantee the full optimization of IVIG. Reports on successful IVIG usage in SFTS cases are limited, although studies have reported a regimen of 0.5 mg/kg/day for 2 days in a child and 1 g/kg 24 h quantity divided into three doses for 3 days in two adult patients (9, 30). In this study, our findings suggested that higher dosages (≥80 g) and a prolonged duration of IVIG treatment may improve the prognosis of SFTS patients. Specifically, for every 1 g increase in IVIG dosage, the risk of death in SFTS patients falls by approximately 1.9% on the 28th day after the commencement of IVIG treatment. This finding is valuable for guiding clinical practice. The pharmacokinetic effect of IVIG on SFTS patients still requires further exploration to verify the benefits of higher IVIG dosages. The half-life of IVIG is approximately 18–32 days and the increase in IgG concentration (ΔIgG) after IVIG infusion has recently been identified as a determining factor in the outcomes of neurological disorders such as Guillain–Barré syndrome and multifocal motor neuropathy (31, 32). IgG is a primary component of IVIG and is responsible for immune-modulating effects, while immune deficiency is a leading cause of severe SFTS (33). Thus, to optimize IVIG usage, further pharmacokinetic monitoring of serum IgG levels after IVIG treatment should be conducted in SFTS patients. Several studies have confirmed that high-dose IVIG therapy is necessary to achieve anti-inflammatory effects (34, 35). Also, after high-dose immunoglobulin infusion, patients showed a stepwise increase in serum IgG levels that were sustained much longer than with low-dose immunoglobulin infusion (36). In clinical practice, SFTS patients usually present neurological symptoms during the MODS period, so we believe that a high IVIG dosage through prolonged IVIG treatment may optimize the effect of IgG and prevent cytokine storms during this stage (37, 38). Thus, it may be beneficial if physicians increase the overall IVIG dosage by extending the duration of IVIG treatment in patients with neurological symptoms.

We recognize that there are several limitations to this study. First, this is a retrospective study with a relatively small patient sample size. Therefore, well-designed large-scale studies are still required to validate our findings. Besides, tests related to encephalitis/encephalopathy, such as the CSF test and electroencephalogram, were not conducted on all our patients. Thus, the relationship between neurological complications and encephalitis still needs further validation. Moreover, we did not evaluate dynamic changes in cytokines during IVIG treatment. Identifying changes in cytokine levels or immunomodulatory cells may help determine therapy initiation and duration. Finally, this study is a single-center study, and the results are limited.

## Conclusions

In summary, SFTS patients with neurological symptoms in our study had a higher mortality rate compared with other studies. Thus, it is necessary to improve the treatment approach for SFTS patients with neurological symptoms. IVIG therapy is a valuable technique that has a favorable impact on the treatment of SFTS with neurological complications. According to our study, a higher dosage through the prolonged application of IVIG treatment offers a good prognosis for patients suffering from SFTS with neurological symptoms. Therefore, we believe it is preferable to increase the overall dosage by extending the duration of IVIG treatment. Further clinical and immunological studies should be performed to enhance our understanding of how IVIG therapy works in SFTS patients with neurological symptoms.

## Data availability statement

The raw data supporting the conclusions of this article will be made available by the authors, without undue reservation. 

## Ethics statement

The studies involving human participants were reviewed and approved by Ethics Committee of Nanjing Drum Tower hospital. Written informed consent for participation was not required for this study in accordance with the national legislation and the institutional requirements.

## Author contributions

YL and CJ contributed to conception and design of the study. HT and YZ organized the database. CJ and FH performed the statistical analysis. YL wrote the first draft of the manuscript. CW and JW wrote sections of the manuscript. All authors contributed to the article and approved the submitted version. 
