# Corrigendum: Effect of intravenous immunoglobulin therapy on the prognosis of patients with severe fever with thrombocytopenia syndrome and neurological complications

**DOI:** 10.3389/fimmu.2024.1383797

**Published:** 2024-04-05

**Authors:** Yun Liu, Hanwen Tong, Fei He, Yu Zhai, Chao Wu, Jun Wang, Chenxiao Jiang

**Affiliations:** ^1^ Department of Emergency Medicine, Nanjing Drum Tower Hospital, The Affiliated Hospital of Nanjing University Medical School, Nanjing, China; ^2^ Department of Infectious Disease, Nanjing Drum Tower Hospital, The Affiliated Hospital of Nanjing University Medical School, Nanjing, China; ^3^ Department of Pharmacy, Nanjing Drum Tower Hospital, The Affiliated Hospital of Nanjing University Medical School, Nanjing, China

**Keywords:** intravenous immunoglobulin, mortality, severe fever with thrombocytopenia syndrome, neurological complications, dosage, duration

In the published article, there was an error in **Figure 2** as published. The unit of IVIG dosage was displayed as “mg”. The corrected Figure 2 and its caption Comparison of (A) IVIG dosage and (B) IVIG duration between the survival group and the death group. **P <0.01. appear below.

**Figure 2:**
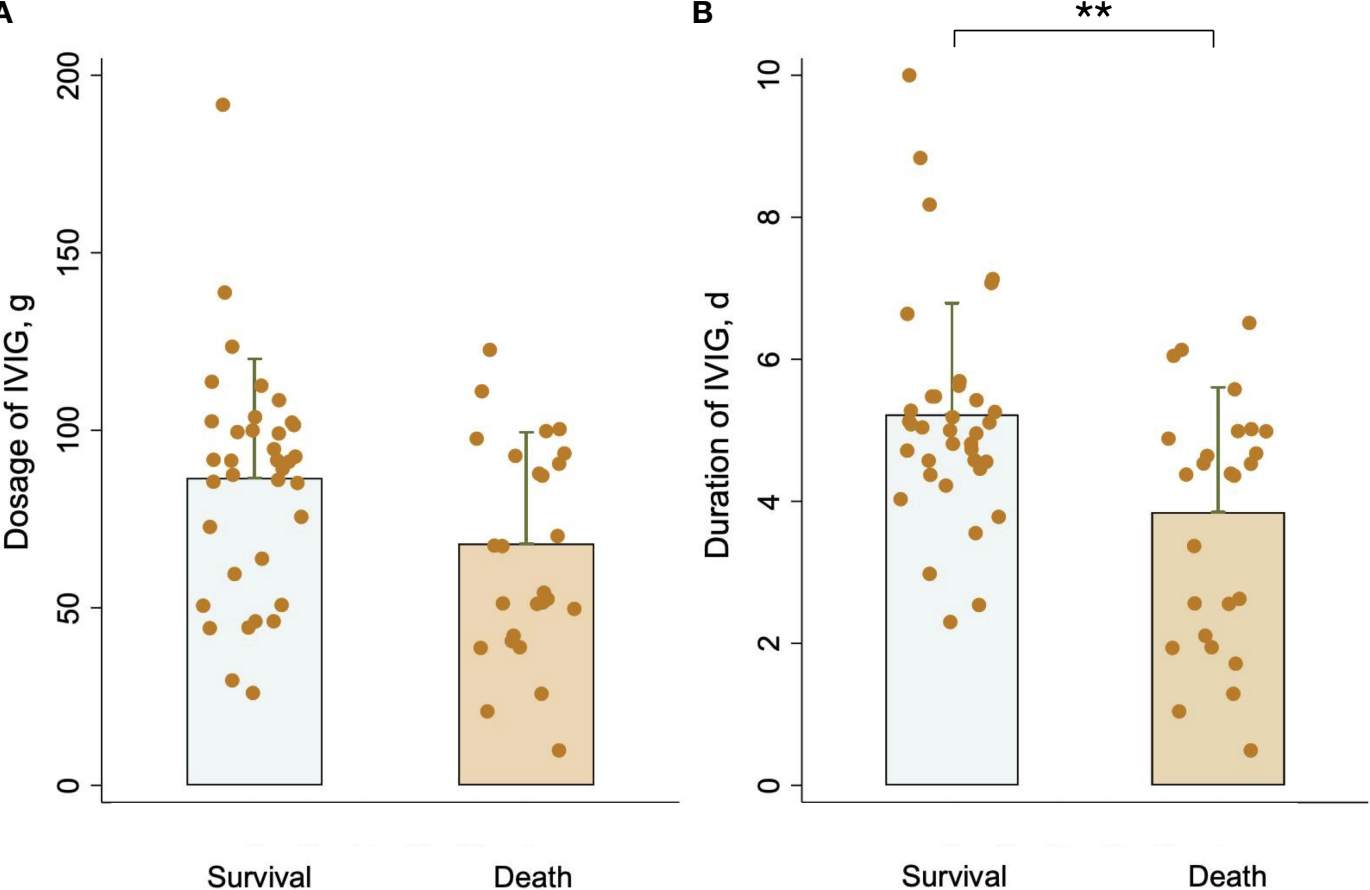
Comparison of **(A)** IVIG dosage and **(B)** IVIG duration between the survival group and the death group. **P <0.01.

In the published article, there was an error in **Figure 4** as published. The unit of IVIG dosage was displayed as “mg”. The corrected Figure 4 and its caption Kaplan–Meier curves estimating 28-day mortality in SFTS patients based on (A) IVIG dosage and (B) IVIG duration. appear below.

**Figure 4:**
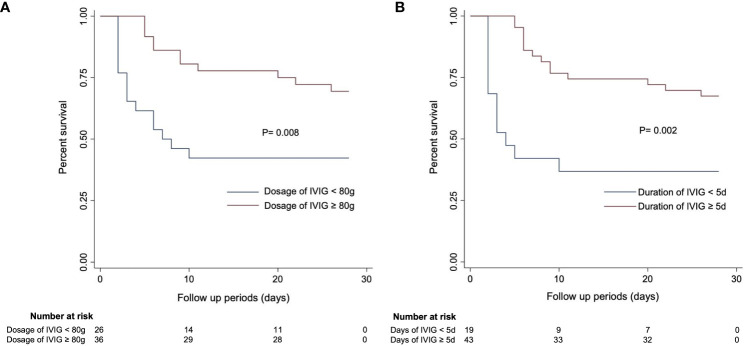
Kaplan–Meier curves estimating 28-day mortality in SFTS patients based on **(A)** IVIG dosage and **(B)** IVIG duration.

In the published article, there was an error in **Table 1** as published. The unit of IVIG dosage was displayed as “mg”. The corrected Table 1 and its caption Baseline clinical characteristics and laboratory parameters of patients in the survival and death groups. appear below.

**Table 1:** Baseline clinical characteristics and laboratory parameters of patients in the survival and death groups.

Variable	Survival (n = 36)	Death (n = 26)	*P*-value
**Demographics**			
Age, years	67 (54-73)	71 (58-76)	0.199
Male, n (%)	18 (50.0%)	11 (42.3%)	0.549
**Chronic comorbidities, n (%)**			
Hypertension	11 (30.6%)	10 (38.5%)	0.516
Diabetes mellitus	3 (8.3%)	2 (7.7%)	1.000
Malignancy	1 (2.78%)	0 (0%)	1.000
CAD	1 (2.78%)	0 (0%)	1.000
COPD	0 (0%)	1 (3.85%)	0.419
**Clinical manifestations, n (%)**			
Nausea	12 (33.3%)	7 (26.9%)	0.589
Vomiting	10 (27.8%)	9 (34.6%)	0.564
Celialgia	4 (11.1%)	2 (7.7%)	0.653
Diarrhea	17 (47.2%)	10 (38.5%)	0.492
Unintelligible speech	1 (2.8%)	3(11.5%)	0.300
Dizziness and headache	18 (50.0%)	6 (23.1%)	0.032
Cough	9 (25.0%)	4 (15.4%)	0.359
Sputum	7 (19.4%)	3 (11.5%)	0.627
Chest tightness	3 (8.3%)	3 (11.5%)	0.689
Rash	6 (16.7%)	1 (3.8%)	0.222
Lymphadenopathy	13 (36.1%)	7 (26.9%)	0.445
Bleeding spots on the skin	7 (19.4%)	9 (34.6%)	0.178
**Laboratory parameters**			
WBC count, median (IQR), ×10^9^/L	2.5 (1.8-4.6)	2.7 (1.7-3.7)	0.898
ANC count, median (IQR), ×10^9^/L	1.7 (1.0-2.9)	2.1 (1.2-2.8)	0.668
ALC count, median (IQR), ×10^9^/L	0.7 (0.4-0.9)	0.5 (0.3-0.6)	0.042
NLR, median (IQR)	2.2 (1.5-6.1)	3.3 (1.9-9.1)	0.136
RDW, median (IQR), %	13.2 (12.8-13.5)	13.6 (12.8-14.3)	0.071
PLT count, median (IQR), ×10^9^/L	44.5 (33.8-61.3)	39.5 (29.0-58.5)	0.480
PLR, median (IQR)	65.5 (39.3-146)	89.6 (67.5-145.0)	0.248
PT, median (IQR), s	12.0 (11.2-12.6)	12.4 (11.7-13.0)	0.123
APTT, median (IQR), s	41.4 (33.6-46.3)	48.5 (37.6-61.1)	0.009
TT, median (IQR), s	22.8 (21.1-27.7)	26.9 (21.8-57.9)	0.057
D-dimer, median (IQR), mg/L	3.8 (2.3-9.2)	9.9 (3.4-22.6)	0.018
ALT, median (IQR), U/L	69.6 (54.3-97.2)	75.3 (50.5-90.5)	0.881
AST, median (IQR), U/L	172.5 (93.4-312.9)	222.0 (114.1-321.8)	0.304
ALB, median (IQR), g/L	32.0 (28.7-35.7)	31.7 (29.1-34.1)	0.775
TBIL, median (IQR), µmol/L	11.0 (8.6-13.9)	7.7 (5.8-11.5)	0.066
SCr, median (IQR), µmol/L	72.5 (48.8-89.6)	82 (67.5-123.6)	0.090
BUN, median (IQR), mmol/L	5.2 (3.5-7.1)	5.9 (4.8-10.6)	0.061
UA, median (IQR), µmol/L	306 (236-347)	342 (231-463)	0.471
**IVIG usage**			
IVIG dosage, g	95.0 (57.5-100.0)	60.0 (40.0-100.0)	0.066
IVIG duration, d	5 ± 2	4 ± 2	0.003
**Additional information**			
Time from onset to arriving in hospital, d	7 ± 3	7 ± 2	0.317
Time from onset to IVIG treatment, d	9 ± 3	9 ± 3	0.677

CAD, coronary artery disease; COPD, chronic obstructive pulmonary disease; WBC, white blood cell; ANC, absolute neutrophil count; ALC, absolute lymphocyte count; NLR, neutrophil-to-lymphocyte ratio; RDW, red cell volume distribution width; PLT, platelet; PLR, platelet-to-lymphocyte ratio; PT, prothrombin time; APTT, activated partial thromboplastin time; TT, thrombin time; ALT, alanine aminotransferase; AST, aspartate aminotransferase; ALB, serum albumin; TBIL, total bilirubin; SCr, serum creatinine; BUN, blood urea nitrogen; UA, uric acid; IVIG, intravenous immunoglobulin; IQR, interquartile range.

In the published article, there was an error. The unit of IVIG dosage was displayed as “mg”.

A correction has been made to **Introduction**, Paragraph Number 161. This sentence previously stated:

“Ultimately, we determined that an IVIG dosage of more than or equal to 80 mg through a prolonged treatment duration of five or more days serves as a good prognosis predictor in SFTS with neurological symptoms.”

The corrected sentence appears below:

“Ultimately, we determined that an IVIG dosage of more than or equal to 80 g through a prolonged treatment duration of five or more days serves as a good prognosis predictor in SFTS with neurological symptoms.”

A correction has been made to **Results**, Paragraph Number 694. This sentence previously stated:

“Patients with an IVIG dosage of more than or equal to 80 mg (Figure 4A) and an IVIG duration of 5 days or more (Figure 4B) had higher survival rates.”

The corrected sentence appears below:

“Patients with an IVIG dosage of more than or equal to 80 g (Figure 4A) and an IVIG duration of 5 days or more (Figure 4B) had higher survival rates.”

A correction has been made to **Discussion**, Paragraph Number 884. This sentence previously stated:

“In this study, our findings suggested that higher dosages (≥80 mg) and a prolonged duration of IVIG treatment may improve the prognosis of SFTS patients.”

The corrected sentence appears below:

“In this study, our findings suggested that higher dosages (≥80 g) and a prolonged duration of IVIG treatment may improve the prognosis of SFTS patients.”

The authors apologize for the errors and state that this does not change the scientific conclusions of the article in any way. The original article has been updated.

