# Supplementary material for: Effect of intravenous immunoglobulin therapy on the prognosis of patients with severe fever with thrombocytopenia syndrome and neurological complications
Source: Front Immunol. 2023 Mar 22;14:1118039. doi: 10.3389/fimmu.2023.1118039 (PMC10073413; doi:10.3389/fimmu.2023.1118039)
Supplement: Supplementary file 1 [file DataSheet_1.docx]

**Effect of Intravenous Immunoglobulin Therapy on the Prognosis of Patients with Severe Fever with Thrombocytopenia Syndrome and Neurological Complications**

**Yun Liu^1†^, HanWen Tong^1†^, Fei He^1†^, Yu Zhai^1^, Chao Wu^2*^, Jun Wang^1*^, ChenXiao Jiang^3*^**

^1^Department of Emergency Medicine, Nanjing Drum Tower Hospital, The Affiliated Hospital of Nanjing University Medical School, Nanjing, 210008, China

^2^Department of Infectious Disease, Nanjing Drum Tower Hospital, The Affiliated Hospital of Nanjing University Medical School, Nanjing, 210008, China

^3^Department of Pharmacy, Nanjing Drum Tower Hospital, The Affiliated Hospital of Nanjing University Medical School, Nanjing, 210008, China

*** Correspondence:**

ChenXiao Jiang

[sharejcx@163.com](mailto:sharejcx@163.com,)

Jun Wang

[wjgaogou@aliyun.com](mailto:Wjgaogou@aliyun.com)

Chao Wu

[dr.wu@nju.edu.cn](mailto:dr.wu@nju.edu.cn)

**^†^** These authors contributed equally to this work

**Supplementary Figures and Tables**

**
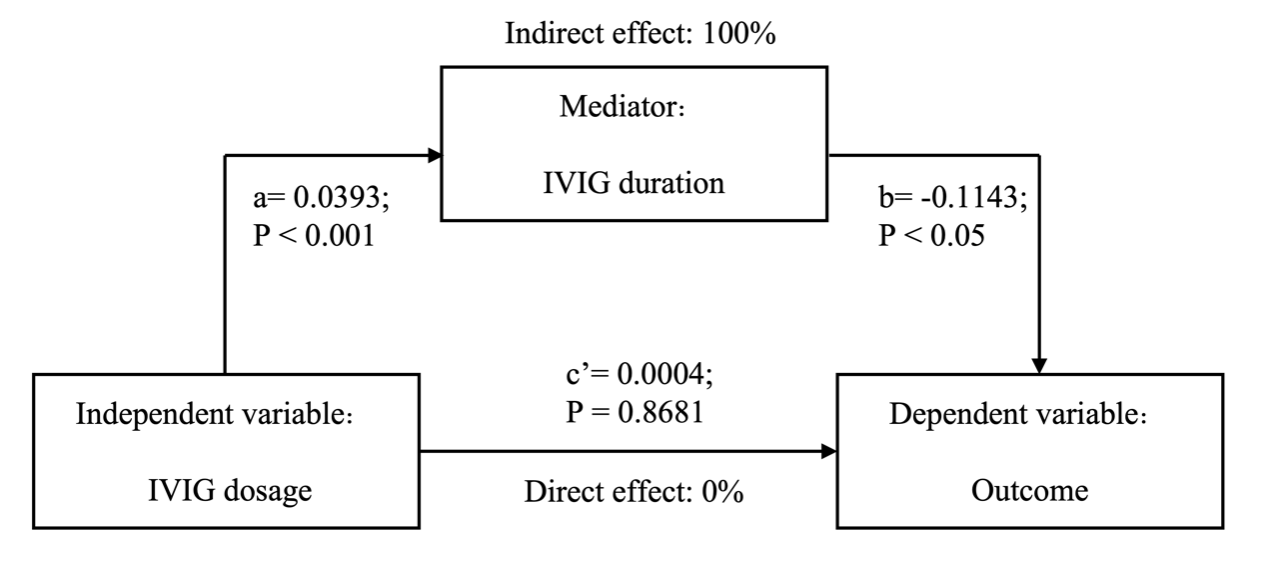
**

**Supplementary Figure 1** Mediation analysis results (including standardized regression coefficients and associated P-values; relative contributions of the indirect and direct effects to the total effect)**.** IVIG, intravenous immunoglobulin.

**Supplementary Table 1** Therapies other than IVIG of SFTS patients in the survival and death group.

| **Therapies** | **Survival**  **(n=36)** | **Death**  **(n=26)** | **P-value** |
| --- | --- | --- | --- |
| Antibacterial therapy | 32 (88.89%) | 24 (92.31%) | 1.000 |
| Antifungal therapy | 15 (41.67%) | 12 (46.15%) | 0.725 |
| Corticosteroid therapy | 14 (38.89%) | 17 (65.38%) | 0.039 |
| RhG-CSF therapy | 25 (27.30%) | 22 (19.70%) | 0.169 |
| Plasma exchange | 1 (2.78%) | 4 (15.38%) | 0.152 |
| Blood transfusion | 21 (58.33%) | 25 (96.15%) | 0.001 |
| CRRT | 1 (2.78%) | 3 (11.54%) | 0.300 |
| Oxygen inhalation | 22 (61.11%) | 23 (88.46%) | 0.017 |
| Respiratory support | 2 (5.56%) | 9 (34.65%) | 0.005 |

IVIG, intravenous immunoglobulin; RhG-CSF, recombinant human granulocyte colony-stimulating factor; CRRT, continuous renal replacement therapy. Respiratory support included noninvasive or invasive ventilation.

**Supplementary Table 2** Mediating effect of duration of IVIG on the relationship between dosage of IVIG and outcome (n = 62).

| **Parameter** | **Coefficient** | **SE** | **P-value** |
| --- | --- | --- | --- |
| a- Relationship between dosage and duration of IVIG | 0.0393 | 0.0045 | 0.0000 |
| b- Relationship between mediator (duration) and outcome | -0.1143 | 0.0504 | 0.0234 |
| a*b- Indirect effect | -0.0045 | 0.0020 | 0.0283 |
| c’- Direct effect | 0.0004 | 0.0027 | 0.8681 |
| c- Total effect | -0.0040 | 0.0018 | 0.0274 |

IVIG, intravenous immunoglobulin; SE, standard error (SE).
